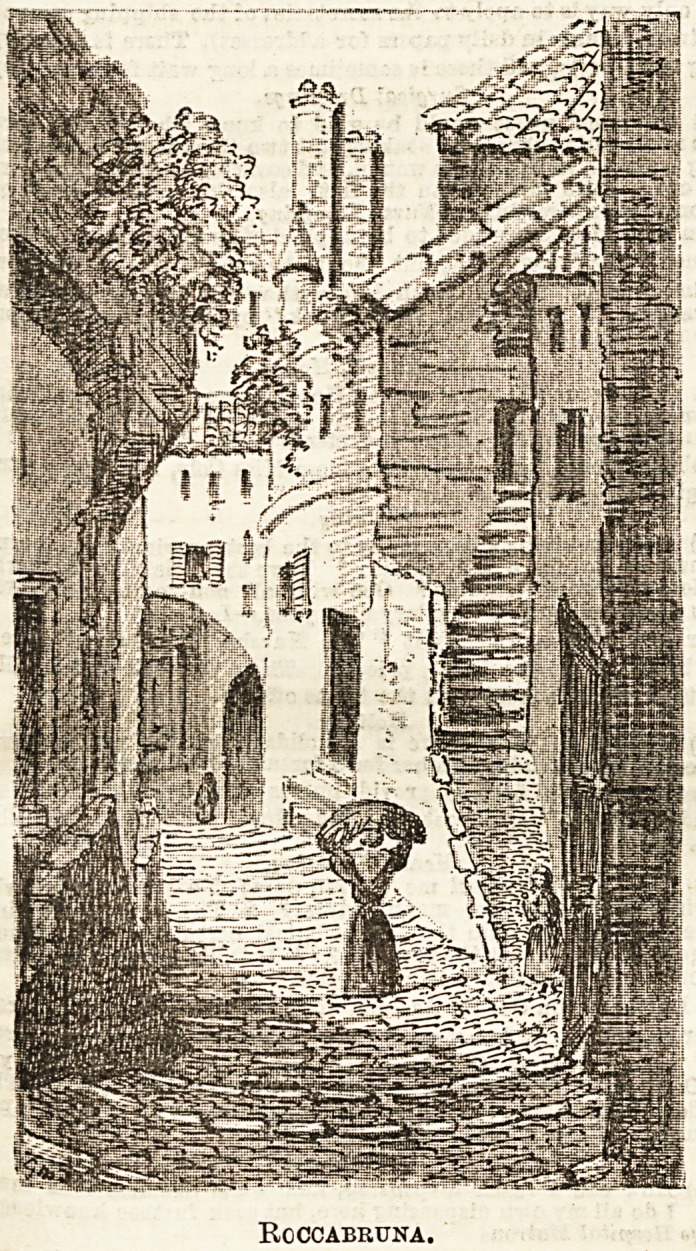# "The Hospital" Nursing Mirror

**Published:** 1899-02-04

**Authors:** 


### "The Hospital" Nursing Mirror.

Being the Nursing Section of "The Hospital."

[Contributions for this Section of "The Hospital" should be addressed to the Editor, The Hospital, 28 & 29, Southampton Street, Strand, London, W.C., and should have the word "Nursing" plainly written in left-hand top corner of the envelope.]

### News from the Nursing World.

### DEATH OF THE PRINCESS OF BULGARIA.

The death of the Princess Marie Louise, wife of Prince Ferdinand of Bulgaria, just after the birth of her fourth child, comes as a terrible blow to her many friends. The Princess was born in 1870, and was brought greatly into notice at the time of her little son Prince Boris' "conversion" to the Greek Church. She, herself an ardent Catholic, was genuinely distressed at the triumph of political expediency in the matter. Her death was caused by influenza and pneumonia following so closely upon her confinement. She was popular with the Bulgarians, and leaves four children, two sons and two daughters, to mourn her loss. The event has created much sympathy for Prince Ferdinand and her parents.

### THE JUNIUS S. MORGAN BENEVOLENT FUND.

We are glad to learn that during the past year the policyholders in the Royal National Pension Fund for Nurses have become annual subscribers to their Benevolent Fund to a larger extent than in 1897, when the plan of assisting it by shilling subscriptions was first initiated. There is hardly a nurse belonging to the Pension Fund who cannot afford 1s. a year to help her sisters in distress, and yet, small as the amount is to each individual, if each of the 3,000 nurses in the Fund paid but one shilling, £150 would result each year to benefit those requiring assistance. We should like to hear of more institutions following the example of the London Hospital, which makes a collection yearly amongst the nursing staff, whether they belong to the Pension Fund or not. This year £11 14s. 3d. has been thus contributed from the London Hospital to help poor nurses through the Junius S. Morgan Benevolent Fund—a most kindly and generous gift, and a very welcome one. Although the Fund has found munificent friends, no public appeal for support is made, and the work is carried on almost in private, and few know how much is done. On the list of what may be regarded as permanent pensioners are nearly forty nurses, who but for the aid so given would have to seek the shelter of the workhouse. Not all of these are members of the Pension Fund, though each year it is found more necessary to restrict the distribution of funds to policyholders, to so many nurses comes a time when through want of work or illness a temporary difficulty in paying premiums arises. Then numbers of nurses have been assisted to join their friends abroad, or have been sent to a suitable climate for their health and have returned well and vigorous to their work. We cannot here enumerate all the various forms in which the Benevolent Fund can and does lend a helping hand; but we have seen numbers of letters from those who have been assisted saying, and with truth, that they can never be grateful enough for the benefits it affords in time of need. It is a splendid feature in the scheme of the Pension Fund, and the more nurses learn to regard it from a co-operative point of view, as belonging to them, the better will it be for them and for the Fund too. It is managed by nurses for nurses, as the committee, which sits every quarter, is composed nearly entirely, of hospital matrons who attend regularly and examine the case of each individual applicant. The most experienced and kindly consideration is thus paid to the applications by those who know the circumstances trials, and difficulties of a nurse's career.

### LONDON TEMPERANCE HOSPITAL.

A very enjoyable entertainment was provided for the nursing staff of the London Temperance Hospital on Wednesday, January 18th. A dramatic performance was got up by Mr. Sanderson and a number of his friends, with a result deserving of all praise. Two short plays were acted, "A Sheep in Wolf's Clothing," and the ever-amusing "Ici on parle Français," both of which were immensely appreciated by the audience. Miss Lucas entertained her visitors to tea in the board-room between the acts, and all present agreed in hearty congratulations upon the success of the evening's amusements.

### "CHILDHOOD."

We draw our readers' attention to a series of lectures now being given at the Sanitary Institute, under the auspices of the Childhood Society. Although intended primarily for teachers the instruction given is invaluable for nurses, especially for those who devote themselves to sick children. Dr. Warner's lecture last night, which taught how to trace what have hitherto been all lumped under the heading "mental defects" to their true source, and how to detect signs of mental fatigue, was valuable to all, but more especially so to those upon whose trained observation the mental and physical health of their patient may depend.

### AN APPEAL FOR INDIA.

In consequence of the development of the work of the Up-country Nursing Association for Europeans in India, and the demand for additional nurses, the committee have issued a special appeal for more support, which has been signed by H.R.H. the Duchess of Connaught. Twelve nurses are at present working for the association, and the Punjaub now requires two more; the North-west Provinces, one now and one later; whilst Madras wants three, so that seven nurses altogether will be required. The engagements are for five years, with a further optional term of three years. Outfit and passages are provided, and the first year's salary is guaranteed by the parent society. The salary is Rs. 75 a month, with board, lodging, and attendance. The hon. secretary is Major-General J. Bonus, The Cedars, Strawberry Hill, W.

### THE NURSES' NEEDLEWORK GUILD.

The result of the Nurses' Needlework Guild, organised a little more than a year ago by Nurse Theobald, of Hatfield, is excellent. The second distribution of clothing has just been accounted for. It amounted to nearly 500 garments, which were sent in parcels of 50 to the Metropolitan Hospital, East London Hospital for Children, the Royal Free Hospital, the Clapham Maternity Hospital, St. Mary's, Paddington, and University College Hospital, whilst the London Hospital and Nazareth House got 60 apiece, and St. Thomas's Hospital 55.

### A BOLD COURSE OF ACTION.

The whole staff of nurses at the Altrincham Hospital were dissatisfied, and not only were they dissatisfied but they expressed their discontent in a letter to their committee. They went still further. They demanded, on pain of immediately resigning their posts, that certain alterations should be made in the arrangement of the hospital. This bold course of action was fortunately somewhat modified upon meeting the committee, and upon learning that it was not possible for them to dictate to their superiors in this high-handed manner. Thereupon they consented to withdraw their letter, and received a promise that a sub-committee should be appointed to inquire into their grievances. The matter has ended with mutual satisfaction, the sub-committee having found that their discontent was well grounded, and having granted their request. Therefore the Altrincham nurses are to be congratulated on the fortunate issue of their adventure. To ask for an inquiry into grievances is one thing; to demand a specified redress under a threat is another; the one is constitutional and orderly, the other is flat rebellion.

### BAZAAR AT LEWES.

The bazaar held in the Corn Exchange, Lewes, last week on behalf of the funds of the Lewes and District Nursing Association was very successful. Lady Chichester opened the proceedings, and said that she hoped that at the top of the report for 1899 there would be written in large letters "Balance in hand." A brisk trade was carried on during the two days' sale, which was rendered more attractive by the fancy dresses worn by the stallholders. The total net gain to the association was £300. It has long been the desire of the committee to provide a home for the district nurses at Lewes, but, unfortunately, there were so many other objects to attract the Jubilee commemoration offerings that the project has not taken shape as yet. But it has not been allowed to drop, and it is hoped that in a short time something more may be done to achieve this end.

### NURSING SCHOLARSHIPS.

A subject of great importance to nurses, but one which receives very little attention, has lately been brought somewhat prominently before our notice. Take for instance the discussion of the Technical Instruction Committee for Yorkshire upon the resolution that "the County Council make a grant of £200 to be set aside for providing sick nursing scholarships to such an amount and extent as the Technical Instruction Committee may decide." It showed that there were no definite rules to guide the committee, either in the amount of the grants or in the selection of the candidates or in the amount and quality of the training, and that the various members had only vague ideas of how the money was laid out. Similar grants are made by other county councils. And the term "training" is so indefinitely stated that it may include the merest smattering of nursing given by maternity or district nursing charities. This state of affairs is detrimental to the status of trained nursing and to the pockets of trained nurses, as we have more than once insisted. This is so because the cottage nurse will not stay in the groove for which she is educated a moment after her agreement expires. Superintendents trying to keep up the standard of nursing in their associations are confronted with the argument that other committees train on the Holt-Ockley system, therefore why bother about a hospital training? Secretaries of committees seeking a cottage nurse to look after the household, as well as the patient, send such queries as the following, "Can you tell me where I can get a nurse who will undertake the work of the cottage? I have asked at —— Home, and no one is willing to undertake domestic duties," the very question implying a misconception of what is meant by a nurse. It is an excellent thing to grant nursing scholarships; it would be better still if these were restricted to women who were prepared to undergo a three-year course of training at a recognised school.

### ESSEX AND COLCHESTER HOSPITAL.

The new home for the nurses of the Essex and Colchester Hospital is now in use. It is a spacious building connected with the hospital by a covered way, and has been erected in commemoration of the Diamond Jubilee. It is a great improvement upon the old nursing quarters, and each nurse has now her separate bedroom, nicely furnished with new modern suites of oak furniture. The floors are stained, varnished, and strips of carpet are laid at the bedsides and elsewhere. The dining and sitting rooms are large and comfortable, made bright and cosy with pictures, occasional tables, and easy chairs. The building is surrounded by a garden, which is a great boon. The committee are now prepared to accept paying probationers. All particulars may be obtained from the matron.

### FOR THE PERTH SICK.

The fourteenth annual report of the Perth Sick Poor Nursing Society was held recently in the Commissioners' Hall. In describing the growth of the society since its foundation the hon. secretary waxed eloquent, and said that "whereas at first there was only the small number of 45 subscribers, now there were hundreds," whilst "the visits had increased by thousands during the last few years." The society appears a useful and healthy one. The two nurses (Nurses Graeme and M'Colman) have worked hard and successfully. During the year the number of cases in their care has been 430, an increase of 29. Their work is well supported by the residents in the neighbourhood, and the accounts show an income of £333, an expenditure of £276, and a balance in hand of £47.

### SHORT ITEMS.

During the past year the Taunton and Somerset Hospital and the Victoria Nursing Institute have been lighted throughout with electric light.—The opening of the new nurses' home and other buildings at the New Hospital for Women, Euston Road, fixed for the 1st inst., has been unavoidably postponed.—A district nurse will be engaged as soon as possible for Gunnislake, Cornwall. At the public meeting recently held to inaugurate the association, promises of annual subscriptions to the amount of £30 were received. Lady Pearson was suggested as president, and the committee appointed.

### Hints on the Home Nursing of Sick Children.

By J. D. E. MORTIMER, M.B., F.R.C.S., formerly Surgical Registrar, &c., at the Hospital for Sick Children, Great Ormond Street.

(*Continued from page* 179.)

### NUTRIENT ENEMATA — SLEEP — BATHING — BED SORES.

### NUTRIENT ENEMATA.

THE temperature of these should be 100 deg. F., and they are best given with a glass syringe, the nozzle being well oiled, or by allowing them to run in from a funnel connected by rubber tubing to a soft catheter passed into the bowel. The lower bowel must be previously emptied by an ordinary soap-and-water enema, if necessary, and the nutrient injections should be given very slowly. Four tablespoonfuls or less, according to age, is enough, as a large quantity will be rejected. Peptonized milk or beef-tea, cream, egg, or Invalid Bovril, with warm water, are suitable. Zyminized nutrient suppositories are often better tolerated than liquid injections, and save much trouble, but care must be taken to see, by inspection of the motions, that they do not remain unabsorbed in the rectum.

### SLEEP.

When in charge of an infant the nurse should see that the face is not too much covered. When this is the case any change may escape her observation; also impure air is breathed in, and there is appreciable risk of suffocation by slight alteration of position. If evacuations are passed unconsciously a sanitary sheet should be used if possible to protect the bed instead of mackintosh. Too much weight of bedclothing should be avoided by doubling the blankets at the lower end rather than at the upper, and by using an eider-down quilt if more warmth is needed. If the bed-clothes are thrown off they should be attached to the head of the bedstead by clips and the child dressed in a flannel sleeping suit or long nightgown pinned below the feet. Fleas, which are often derived from domestic pets, may cause much irritation, and in some children excite an urticarial eruption. Freshly-powdered fennel seed sprinkled in the bed is a good preventive. Underlinen and sheets must, of course, be changed frequently, and it may be also necessary to change blankets, carpets, and mattresses every fortnight or so for a time, as the insects breed in such things and take about a month to fully develop from the eggs. Besides the usual causes of sleeplessness infants and young children are apt to be kept awake by any novel appearance in the room which they do not understand, such as the flickering shadows thrown on the ceiling by a fire. A child should never be woke up if this can possibly be avoided, and in any case it must be done very gently.

### BATHING.

The temperature of an ordinary bath for an young infant or weakly child should be 90 deg. to 95 deg. Fahr. In adding hot or cold water care must be taken to mix it thoroughly with that which is already in the bath before taking the temperature. This may be roughly estimated by the elbow (not the hand), but it is better to use a thermometer, and this must always be done if a hot or cold bath is needed. Bath thermometers are not always correct, and should be tested by putting a clinical thermometer into the same water at 95 deg. to 105 deg., so that errors may be allowed for. If the room is cold or draughty the bath must be given in front of a fire with a screen round. A plaything of china or rubber, or a cork with feathers stuck in it will often save a fit of crying, and if there is likely to be alarm from the steam a blanket should be spread over the bath and the child gradually lowered into the water. Great care must be taken to thoroughly dry the folds about the ears, neck, armpits, navel, and groins; some boracic or zinc powder should be used, and a little absorbent cotton tucked in if necessary. Chafing may also arise from the irritation of discharges or from the diapers being too stiff or having been washed with some coarse soap or much soda, or from a nasty practice of drying them for further use when wetted by urine. If there is much soreness use instead of soap some very thin gruel or oatmeal water (a table-spoonful of oatmeal to a pint of boiling soft water, strain and allow to cool) and apply zinc or boracic ointment.

After feeding (especially weakly babies) the mouth should be cleansed with soft rags (not used twice unless boiled). Older children should have the teeth brushed and rinse the mouth the last thing at night, and at least once during the day. Any "running" or discharge should be taken up by soft rag or absorbent wool and burnt at once—on no account use a sponge. Care must be exercised lest others become contaminated by towels, pinafores, &c.

Pediculi (lice), although usually found in neglected children, may be unexpectedly discovered in others, as a result of riding in public conveyances, &c. If the hair cannot be clipped, the child should lean the head backwards or sideways over a basin (so that nothing goes into the eyes) and the hair should be soaked thoroughly with common kerosene oil or with Izal (a tablespoonful to one pint of water), and afterwards well washed and a fine comb used for the next fortnight. This will kill the nits, although they may still be found sticking to the hair.

As regards application of heat and cold and the employment of counter irritation, it must always be remembered that the skin of a child is delicate and the whole system more sensitive than that of an adult. For instance, hot bottles must be wrapped in several thicknesses of flannel or a blanket; mustard diluted with three or four times as much linseed for a poultice; cold packs and baths given with more caution, and so on.

In most cases 98 deg., raised to 100 deg., is high enough for a hot bath. If mustard is ordered use one to two table-spoonfuls for each gallon of water—it should be made into a paste, and then stirred in. The child should remain in the bath three minutes. For cold sponging, which is often ordered, the child should be placed on a blanket with water-proof underneath it, and be lightly covered by another blanket. One limb at a time should be sponged and dried, tepid water (85 deg.) being used at first. (Use of ice-bag will be noticed in forthcoming papers.) A hot bottle may be put to the feet.

### BED SORES

must be guarded against by keeping the under-sheet or draw-sheet tight and clean, changing the position from time to time when possible, and carefully cleansing and drying the skin, afterwards applying boracic or zinc powder. If there is frequent wetting, &c., zinc ointment may be found to protect the skin better, and some arrangement must be made with a bag of peat-moss, absorbent wool, or a sanitary sheet. The use of mackintosh should be avoided as far as possible. Pressure may be taken off bony prominences by water-beds or cushions, or by rings made of twisted tow or cotton-wool and covered, or by so arranging pillows or mattresses that the part—for instance, the heel—is not in contact with anything at all. If the child has to lie in the prone position on account of threatened sore or other reason, the bedstead should be turned so that he does not have to look at the wall.

*(To be continued.)*

### Training in the Provinces.

(*Continued from page 182.*)

### WOLVERHAMPTON AND STAFFORDSHIRE GENERAL HOSPITAL (230 Beds).

### Terms of Training.

Probationers are received for three years' training at the Wolverhampton and Staffordshire General Hospital, Wolverhampton. Candidates should be between 22 and 30 years of age, and they are required to enter in the first instance upon a trial period of three months before final acceptance as probationers. No salary is given during the first year, payment beginning the second year at £15 per annum, and rising to £20 for the third year, when probationers are promoted to be "assistant nurses." Sisters' salaries range from £27 to £30 per annum. Laundry and indoor uniform are provided by the hospital for all the nursing staff. Lectures are given during the period of training by members of the honorary and resident staffs, and certificates granted at the completion of the three years' engagement. A three years' certificate of training and some experience in the management of wards are essential qualifications for the post of ward sister. Ordinary probationers are eligible for such promotion, but they must have completed their three years, and have taken holiday work for head nurses.

No night duty is taken by probationers in their first year. During the second and third years nurses take night duty for not more than three months consecutively.

### Hours On and Off Duty.

Probationers are on duty between 7 a.m. and 9 p.m., with time for meals to be deducted, and two hours daily for recreation. Days off are given occasionally. Staff nurses' hours are the same, with extra time off duty once a week from 4 p.m. to 9.30 p.m. Sisters have leave of absence every other day from 4 to 9.30 p.m. Three weeks' annual leave is given.

### Meals.

All meals except lunch are served in the dining room, that meal being taken in the ward kitchens. The only weekly allowances made are tea and sugar.

Nurses and probationers breakfast at 6.30 a.m., lunch is at 10 a.m., dinner at 12 and 12.30, tea at 4 30 p.m., and supper at 9. Sisters breakfast at 7.30, their dinner is at 1.30, tea at 4, and supper at 9 p.m.

---

### GENERAL INFIRMARY, WORCESTER (127 Beds).

### Terms of Training.

Two classes of probationers are received for training at the Worcester General Infirmary, those in the one entering for three years, paying a premium of £10 10s., with no salary for the first year, payment beginning at £5 the second year and increasing to £10 for the third year; those in the other receiving a course of two years' training, paying a premium of £15 15s., and being unpaid. The three years' probationers are provided with uniform by the hospital, the others are required to find it for themselves. Sisters' salaries range from £25 to £32 per annum. Probationers are eligible for promotion to the post of ward sister after two years' training if their work shows evidence of their fitness. Night duty is taken by the probationers for from three to four months at a time, under the supervision of a permanent night sister.

### Hours On and Off Duty.

Probationers are on duty in the wards between 7 a.m. and 8.15 p.m., with time for meals, twenty minutes for dressing during the morning, and "off duty" twice a week for one and a half hours, twice for two hours, and twice for three hours. Sisters are on duty from 8 a.m. to 8.45 p.m., with time for meals. They are off duty twice a week for one and a half hours, twice for two hours, and twice for four hours. On "days off," nurses are not on duty at all.

### Meals.

The only meals not served in the dining-room are lunch and tea, which the nurses have in the nurses' sitting-room of each ward. Weekly allowances of tea, sugar, and butter are made to the nurses. The hours for meals are as follows: Probationers' breakfast is at 6.30 a.m.; lunch, 9 to 10; dinner, 1 and 1.30; tea, 4 and 5 p.m.; and supper at 8.15. Sisters' breakfast at 7.40 a.m., their dinner is at 12.45, and supper at 7.30. The hours for lunch and tea are the same as those for probationers.

---

### Minor Appointments.

Queen Anne School, Caversham, near Reading.—On December 12th last Miss Letitia Black was appointed Sister of this institution. She was trained at the London Hospital, where she was afterwards staff nurse of "Crossman." Since then she has been district nurse under the Cathedral Nursing Association, Newcastle-on-Tyne, for five years, and night superintendent of the Royal Berks Hospital, Reading, for three years.

Isolation Hospital, Upney, Barking, Essex.—Miss Phillips has furnished us with fuller particulars of her training. She was appointed Matron of this hospital on the 17th ult. She was trained at Sir Patrick Dun's Hospital, Dublin, and was afterwards for some time superintendent of nurses at Jervis Street, Hospital, Dublin. She has been for the past two years sister at the Borough Hospital, Wolverhampton.

Kasr-el-Ainy Hospital, Cairo.—Miss Margaret Samson, who was appointed Sister at the Kasr-el-Ainy Hospital, Cairo, in November last, was trained under the Nightingale Fund, St. Thomas's Hospital, where she remained three years. She afterwards joined the Queen Victoria Jubilee Nursing Association, for whom she worked as district nurse several years.

South Shields Union Workhouse Infirmary.—Miss Elizabeth Clements was elected Night Superintendent of this infirmary on the 19th ult. She was trained at the Mile End Infirmary, London, E., and was afterwards charge nurse at the Poplar Stepney Sick Asylum District, and at the Brook Fever Hospital.

Allt-yr-yn Fever Hospital, Newport, Monmouthshire.—Miss Gordon-Duguid was appointed Sister here on January 24th. She was trained at the Royal Hospital for Sick Children, Aberdeen, and she has been charge nurse for 14 months of a scarlet fever block of the Northern Hospital, Winchmore Hill.

Woolwich Union Infirmary.—Miss Maude Foster was appointed Head Nurse here on January 5th. She was trained at St. George's Infirmary, Fulham, and has been head nurse at Camberwell Infirmary.

Weymouth Union Workhouse.—Miss Helena Pattison was appointed Superintendent Nurse of this institution on January 24th. She was trained at Bradford, and has been working in Bradford and Sulcoates.

St. Olave's Union Infirmary.—We are informed by Mrs. Emily Groome that in the notice of her appointment last week as charge nurse of the above infirmary her name was erroneously given as Miss Mary Froome.

### Nursing in Enteric Fever.

By Warren G. Westcott, L.R.C.P.Lond., M.R.C.S.Eng., Resident Medical Officer, Chichester Infirmary.

(*Continued from page* 180.)

Diarrhœa: More than three stools in the twenty-four hours should be looked upon as constituting diarrhœa and calling for treatment. Many cases of enteric fever run their entire course without this being at all a leading symptom; indeed, quite the opposite, constipation may be a prominent feature. Diarrhœa may often be traced to the passage of some irritating material along the alimentary tract; the first thing, therefore, to be done is to examine the stools, and in many cases curds will be discovered. This being so, the milk must be diminished in quantity and treated with some diluent, also beef-tea and similar preparations should be discontinued, the extractives which they contain being prone to aggravate the state of affairs present, perhaps sometimes to set it up. The patient should be kept as quiet as possible, bodily and mentally. If, notwithstanding these precautions, it continues, the starch and opium enema should be given, or opium by the mouth, as pil plumbi c. opio for adults, and small doses of T. opii in chalk mixture in the case of children.

Constipation, as mentioned, is often marked throughout the case. It should be relieved by a simple enema when there has been no action of the bowels for four days. Refusal of food is common in young children, and as it is extremely important that sufficient is taken, and at the same time all undue excitement and struggling avoided which its compulsion necessarily entails, nasal feeding should be resorted to. To the nurse possessing the most rudimentary knowledge of the anatomy of the naso-pharynx the passage of the nasal tube can present no difficulties. To prevent any accident she should not introduce food till the child has breathed freely for a minute or so, thereby knowing that the tube has not passed into the larynx; to avoid introducing air into the stomach constrict the tube near the nostril, fill up the funnel, and when all air has escaped allow the fluid to pass onwards. As the use of this causes a certain amount of irritation to the nasal passages, food should be given less frequently, and in therefore larger amounts.

Troublesome stomatitis not infrequently occurs in children, and is best treated with glycerine and borax; for the painful fissures which may form at the angles of the mouth, ung. hydrarg. ammon. dil. answers well.

The more serious states which may arise usually do so at the beginning of the third week and onwards; they may be general as pneumonia, and local as hæmorrhage, peritonitis, tympanitis, perforation, or blood, may appear in the stools in the form of clots, bright or dark, and of various sizes, or it may instead give merely a red colour to the contents of the bedpan, in some other cases it imparts a tarry look to the stool; when occurring early in the case, say within the first fortnight, it is usually small in amount, and is due to extreme congestion of the lymphoid follicles in the wall of the intestine; later it is frequently present in larger quantity, and is due to deep ulceration and separation of sloughs. At this time the utmost care is required of the nurse in the handling of her patient, all unnecessary movement being strenuously avoided. The stimulant, if such is being administered, should be discontinued, unless there be strong indication for its continuance, such as impending syncope. Intestinal astringents as opium, catechu, ergot and the mineral acids are usually given, and cold applied to the abdomen, with the view of contracting the underlying vessels, it may be in the form of an ice poultice or icebag suspended from a cradle beneath the bedclothes, and should be placed over the lower part of the small intestine, that is, in the right iliac fossa. Hæmorrhage may be going on and yet no blood appear in the next stool owing to its retention higher up in the bowel. The nurse receives intimation of any serious bleeding by general pallor, chilliness of the body, fall of temperature, perhaps 2 or 3 deg. or more, increased weakness and rapidity of pulse, quickened respiration, and great restlessness.

Peritonitis: This does not always mean that perforation has occurred, though usually indicative of such. It may come on quite insidiously and its symptoms remain for a time latent, but usually there is an exacerbation of the abdominal symptoms. The pain is generally more severe and tenderness more evident, distension increases, and is due to the filling of the paralysed bowel with gas (tympanitis). Sickness is often extreme, the patient tends to lie low in the bed with knees drawn up, expression anxious, evident signs of collapse are shown by the feeble and rapid pulse, shallow hurried breathing, purely thoracic in type, and coldness of extremities. To ease the pain morphia is given hypodermically and warm anodyne applications to the abdomen. Some obtain greater relief from an icebag. Stimulants need to be freely given, and as there may be distressing sickness rectal feeding should be undertaken. If there be much distension of the bowel a turpentine enema may be given.

Perforation is a rare occurrence in children, but one of the most frequent causes of a fatal termination in adults. It rarely happens before the third week is reached. Its symptoms are as already detailed under the head of peritonitis; the latter being usually due to this they may be marked, but generally there is acute abdominal pain or exacerbation of pre-existing pain, marked tenderness, and distension of the abdomen, the note being everywhere hyper-resonant owing to the presence of free gas in the peritoneal cavity.

Perforation has been attributed in some cases to disturbance of the bowel during defæcation, vomiting, or great restlessness; these undoubtedly predispose. Entire rest must be sought for the bowel, hoping that a local peritonitis will follow and cause agglutination of adjacent coils of intestine, but such a favourable process rarely follows. In the large majority of cases a fatal termination speedily ensues, either from shock or general peritonitis. Pain must be relieved by morphia in large doses hypodermically, and collapse counteracted by general stimulants administered in the same way, or by rectum.

Such may be taken as a general plan of management in cases of enteric fever. Other events may happen which it is needless to mention here. As before stated, in many cases which suffer complications or relapses, the cause is traceable to the too early taking of solid food, including in such apparently harmless articles as bread and butter, bread and milk, and custards; return to these must be made with great caution, and it is therefore a good rule to lay down with regard to their commencement that they are not to be taken until the temperature has been normal morning and evening for ten days, then only sparingly at first; gradually, white fish, chicken, &c., may be allowed. At the time that solid food is commenced the patient may sit up in bed for a short period, and increase the time of so doing daily till sitting up practically the whole day. The bed should not be left till the fourteenth or twenty-first day after normal temperature has been reached. Convalescence is much aided by a visit to some bracing health resort. Plenty of good nourishing food is imperative for restoration to health. A light wine with meals is helpful, and in many cases iron, arsenic, and cod liver oil are useful as general tonics.

### A Book and its Story.

### HER MEMORY.

THE vein of sadness which characterises the writings of Maarten Maartens is not absent in his latest novel.* The scene of the story is laid in England and the characters are treated with an artistic dexterity worthy of the master hand who conceived them. If to the reading public at large it is probable no earlier story of the great Dutch novelist will be more popular; yet to the few, to his more ardent disciples, there is a slight sense of want about it as compared with its predecessors, there is more of the morbid and less of the touching in the plot as in its execution, but "Her Memory" will be a popular novel, if not a work of classic art like "An Old Maid's Love."

Of the woman whose memory Maarten Maartens thus so vividly describes we have but one personal glimpse, and that is at the closing scene of her life: "She lay dying, in the silent summer evening, in the sunlit silence that seems alive with sound. The long shadows deepened round her, through the depths of tranquil sunset, the soft shadows all around her, closing in upon the sunlight of her life. He knew it. He sat beside the bed, his arms fallen between his knees, his face flung forward, intense with straining, as if to draw her back before she slipped away. During ten short years—a moment—she had filled his life with summer; she had been—she was—his sunrise; his day was young yet, young as hers. . . . She opened her eyes and looked at him. 'Anthony,' she said, in a voice like that of a stranger, speaking very low and calm, 'I want you to fetch her, please.' . . . He rebelled against this inevitable desire of hers, the leave-taking from their only child. And he crept away, with laggard step, to the farther side of the house, and took the child's hand from her toys, and brought her." . . . The woman dies, and the husband is left, and the widower's relations to his child form the theme of the story, a theme which perforce lends a tone of sadness to the whole book. Stollard is a man of emotions, for many years he lives on them. He leaves this home property, the home which she had blessed with her presence, and he resolves he will never return; he and the child Margie go abroad.

"Of the dead mother left behind them, the joy dropped from their lives, he never spoke. The child must be happy . . . The child still clung, through memories which daily grew fainter, to the dear image she yearned not to lose. She was eight years old; she could understand as much as most of us about earthly love and death's consummation, the mystery before and the mystery beyond. She longed to recall with her father the one great happiness which already in her young life formed a past."

In the grown man's soul, on the contrary, a vain desire had awakened to forget. Resentment at her leaving him arose in his breast, and resentment mingled with regret made forgetfulness impossible. "She was pure and good, and prayed to God daily. If God hears prayers at all, He must hear such prayers as hers. Had she asked Him, He would have let her remain."

Anthony Stollard and the child Margaret wander from one spot on the Continent to another. At Monte Carlo he unexpectedly meets an old friend, Lady Mary Hunt; the two had been friends in their pre-matrimonial days. Lady Mary's character is described admirably, and it is with a sense of pleasure one turns to her bright and exuberant personality, and Stollard's pleasure equals the reader's. "It must be admitted that, after three months of unadulterated Margie and melancholy he had felt the repellent attraction of Monte Carlo increasing upon him at every minute, when whom should he meet, of all persons, but Lady Mary Hunt! Twelve years ago, when they were little more than children, Lady Mary and he had found unadulterated pleasure in one another's society. In those days she was Lady Mary Dellys, one of the too-numerous daughters of the Stollards' noble but impoverished neighbour, the Earl of Foye. People quite expected to see them 'make a match of it,' when suddenly her engagement was announced to Mr. Thomas Hunt, of the City firm of Hunt, Fenning. . . . It was the old squalid story, and all Lady Mary's relations agreed with her that she had acted for the best, and, on the whole, was lucky. Two years later Anthony, perfectly heart-whole, married a girl he had loved at first sight—and second—and Lady Mary, perfectly contented, sent him a silver butter-dish. And now this woman suddenly crosses his path with her grown-up stepdaughter beside her. In those early days he had always found her delightful to talk to; a healthy element, full of the qualities he lacked—easy good nature, good sense. He was fascinated now by the desire to compare her with herself; the whole of his married life lay between Lady Mary Dellys and Lady Mary Hunt." The two see much of each other, and Anthony Stollard is roused out of his morbid self-concentration by the brilliant woman of the world. Her advice to him on sundry occasions is salutary, if characteristic. "Of course you are miserable," she said once, "everyone can see that in your face . . . and of course one can understand your being miserable after your terrible loss. But mark my word, you are just the man to mix up misery and enjoyment till you don't know which is which. It's the greatest mistake a human being can make; they're both good enough, and right enough, if only you keep them apart. Once mix them up and misery is bound to swallow up everything else. . . . Stop enjoying your sorrow at once. . . . No reasonable man's life is only a love story." Aroused by his old friend's words and by other influences Stollard awoke a little from his melancholy. The love of his art woke too, and with the little Margaret and her governess he settled down in Florence and recommenced his artist life. Like all men of emotion, as opposed to action, Anthony is the victim to advice. Mrs. Fosley, his mother-in-law, perhaps the most lifelike character in the book, writes him urgent letters to return to duties which await him, as a property holder, in England. His closed mansion is going to ruin, she urges, the county was disappointed in Anthony; Sir Henry Stollard, his eldest brother, is leading a great, a useful career; hints which each alike fall unheeded on the artist's ear. Time goes on, and at length the public life of Sir Henry Stollard is cut short by death, and Anthony returns to England, aroused by a stern sense of duty to take up the self-same career which has been ended in his brother's case. Now it is we see him the man of action—a pleasanter study than at our first introduction to him. Margaret grows up in the lovely English home not far from the old house where she was born. "Busy as Anthony's life was now, with a constant inevitable activity of a man before the public, the periods of rest he devoted to his daughter. . . . Sir Anthony was a rising politician, fantastic many thought, and not always sufficiently matter-of-fact, but a man of heart and brain; and Margie was growing into a woman, a serious young creature, over-weighted with loyalty to early traditions and responsibilities towards her father and herself." At this point in the narrative Mr. Hunt dies, and Lady Mary is a widow: but she is not a widow long, for the closing chapter in the story shows Sir Anthony breaking a piece of news to his daughter: "I have asked Lady Mary Hunt to marry me, and she has consented"; and the girl said, "I am glad." So ends the story, and we close the leaves of Maarten Maarten's story with none of the sadness with which we follow it through its early course.

* "Her Memory." Maarten Maartens. (London: Macmillan and Co. 1898.)

### Everybody's Opinion.

[Correspondence on all subjects is invited, but we cannot in any way be responsible for the opinions expressed by our correspondents. No communication can be entertained if the name and address of the correspondent is not given, as a guarantee of good faith but not necessarily for publication, or unless one side of the paper only is written on.]

### HOME FOR NURSES NO LONGER ABLE TO WORK.

"Policy 278" writes: As one of the "First Thousand" I am sadly grieved to find that I have not availed myself of the opportunity of expressing my deep and sincere gratitude for the immense benefit conferred upon nurses through Mr. Burns' generosity and great kindness towards us. I also wish to thank you, the true friend of nurses, for your wise and far-seeing remarks on "A Constant Reader's" letter, "Home for Nurses no Longer Able to Work." Desirable as a home may appear to many minds, I quite agree with you that such would have a decided tendency to irksomeness. To my mind what constitutes the greatest happiness in home life is congenial variety with nobleness of purpose, avoiding grooves as much as possible, and continuing to live for others rather than self.

"M. C. H." writes: As district nurse and subscriber to the R.N.P.F., I trust you will allow me space in your paper for a few words on the subject of a home for nurses past work. As you know, a nurse who has worked twenty years is pretty well used up at 55, and really needs more than the £20, or at utmost £30, a year she has been able to provide by means of the N.P.F., if, as is almost always the case, she has had others to help cut of her slender pay. But, supposing she has not been able to save even that, the objection that women would find the life irksome in a home (although I find governesses thankful for the help such a home gives, even if it is irksome) might be met by providing only house rent, firing and attendance, and allowing each nurse to make a home of her own bed-sitting room. Arrangements for a common mid-day meal could easily be made. But supposing the objection of the irksomeness of such a life is allowed to be great, why could not the scheme provide extra pensions, as is done for governesses, who prefer this life to going into a home?

### Examination Questions for Nurses.

In response to urgent requests we have received we propose to resume the series of monthly examination questions which, partly through pressure on the space in our columns, we had ceased to continue as formerly. The questions set for examination in the new series will be as practical as possible, that observation and thought may both be exercised. The papers, which may be signed with a nom-de-plume, but accompanied by the sender's name and address, will be submitted for expert opinion, and the replies to the questions considered to be the best will be published, and the writer will be offered the choice of a useful book from the catalogue of the Scientific Press. Answers, marked "Examination Questions," must reach the Editor, 28, Southampton Street, Strand, within one fortnight of the publication of the paper.

Question for February.

"What steps would you take to prevent a helpless patient from slipping down in a bed when no appliances were to be had. In a cottage, for instance, where the occupants were very poor?"

Answers must not exceed 600 words.

### Pension Fund Nurses.

### MISS BURNS' WEDDING GIFT.

We have received the following contributions: Nurses M. Roberson (Natal), E. T. Sinclair, and M. A. Rogers.

### Appointments.

Infectious Diseases Hospital, Bow Arrow Lane, Dartford.—Miss A. Talbot was appointed Matron here on January 17th. She was trained at the Wirral Children's Hospital, Birkenhead, and at the Leeds General Infirmary. Miss Talbot has been charge nurse at the Eastern Hospital, Homerton, London, for three years, and for nearly two years night superintendent and deputy matron of the Nottingham City Isolation Hospital.

Huddersfield Sanatorium.—On the 11th ult. Miss A. Milligan was appointed Matron of this hospital. She was trained at Sir Patrick Dun's Hospital, where she remained eight years. Miss Milligan has been sister at the Monsall Fever Hospital, sister at the Fleming Memorial Hospital, Newcastle-on Tyne, temporary matron of the Kidderminster Hospital, and matron of the Wolverhampton Fever Hospital for six years.

The Hospital, Newark-on-Trent.—Miss Elizabeth Wiseman has been appointed Lady Superintendent of this hospital. Miss Wiseman was trained at the Hospital for Sick Children, Shadwell, and at St. John's House, and since August, 1895, has held the post of sister at the Royal South Hants Infirmary, Southampton.

Infectious Hospital, Wimbledon.—Mrs. A. Lockitt has been appointed Matron of the above. She was trained at St. Thomas's Hospital, Westminster, and has since worked under the Metropolitan Asylums Board, first as night superintendent, and for the last two and a half years as assistant matron at the Brook Hospital, Shooter's Hill.

Maternity Hospital, Manchester.—On January 16th, 1899, Miss M. C. Lancaster was appointed matron of this hospital. She was trained at St. Thomas's Hospital, London, and has been sister at the Birmingham Infirmary, and sub-matron and superintendent at the Lying-in Institution, Brighton.

Taunton and Somerset Hospital.—Miss Tearle has been appointed Sister of Blake Ward. She received her training at this hospital. Miss Tregar has been appointed Sister of the William Liddon Ward (children's). She trained at the Children's Hospital, Gloucester, and has since worked as sister at the West Ham Hospital.

Samaritan Hospital, San Paulo, South America.—Miss Nellie Robinson has been appointed Sister at this hospital. She was trained at the Taunton and Somerset Hospital, where she afterwards held the post of sister in the children's ward.

Axminster Cottage Hospital.—Miss Thompson has been appointed Matron of this hospital. She trained for three years at the Taunton and Somerset Hospital, afterwards holding the post of sister of Blake Ward (men's medical).

### Our Convalescent Fund.

We thank "Evelyn" very much for her kind contribution of 1s. 6d. to our Convalescent Fund. We should be glad to acknowledge any number of contributions, as our funds are still too low.

### Wants and Workers.

[The attention of correspondents is directed to the fact that "Helps in Sickness and to Health" (Scientific Press, 28 & 29, Southampton Street, Strand, London, W.C.) will enable them promptly to find the most suitable accommodation for difficult or special cases.]

Will the nurse who wrote at the beginning of January to Miss Taylor, Eland House, Rosslyn Hill, Hampstead, about going out to Marsovan kindly communicate with her, as she has reason to believe her letter in reply has miscarried?

Miss Alice Browne, Chertsey House, Park Hill Rise, Croydon, will be pleased to give last year's Hospital to any nurse unable to buy it for herself.

### Mental Hygiene.

On January 31st Dr. Francis Warner, F.R.C.P., delivered a lecture at the Sanitary Institute, 72, Margaret Street, W., on "Mental Abilities and Disabilities of Children." It was the first of a series arranged by the "Childhood" Society. The paper was a valuable one, and pleaded that mental hygiene should be studied on the same basis as physical hygiene—by observation and experiment. He said that spontaneity of action, easily controlled movement, capacity for games and occupation, and good imitative power in action and speech indicated aptitude, and that it was desirable that all the faculties should be tested separately and collectively. It was often found that supposed inaptitude had nothing to do with the brain at all, but arose from inaccurate observation arising from perhaps some physical defect, and was consequently curable. Brain fatigue arose from many causes besides over-pressure, such as want of exercise, bad ventilation, dirt, &c. The signs of it were easily detected in face and fingers. In such cases the force of controlled movement was lessened, these movements were fewer, whilst uncontrolled movements were more frequent, the child reverting to a more childish stage of spontaneity. There were finger twitchings, contraction of the forehead, unequal balance of the out-stretched arms, with drooping fingers instead of level when in that position, slower and more inaccurate response to commands, lower voice, &c. Mental fatigue may be present and yet good mental work may be done. It is even good to experience some degree of mental fatigue, but it is a condition of danger, and the night's rest should recreate the day's fatigue, the vacation rest recreate the term's work. Passing on to mental confusion, Dr. Warner described the spontaneous movements in the young babe which result from the action of the many detached motor centres in the brain. Until four months of age there was no sign of co-ordinate movement—no power to inhibit spontaneous movement—but at six months of age a child would pause and regard with arrested attention a bright-coloured ball if it were held before it, and the nurse would say, "Baby takes notice." In that pause a new series of co-ordinate thought processes began, the baby, like a general marshalling his army before giving the word of command, so collected its forces, preadjusting its brain centres, and performed an act of attention. Psycologists deny that the will of the child acts, and hold that the external object acts upon it. Teachers should encourage spontaneity as much as possible, and not repress it. Teachers should allow a pause for thought before requiring answers to their questions. The attention of the majority of children is reached through the eye—of a few through the ear. It is better to use one or the other separately rather than both together. It is better to give slight suggestions rather than strong; to speak in a quiet voice rather than in a loud one; to give a hint rather than a command. Simplicity of direction and accuracy of movement, or of any series of movements (as in gymnastics) to be imitated, are necessary to produce a clear impression on the attention of the pupil. Fidgetting, irrelevant answers may be evidences of processes of thought, whilst the "sharp" answers of precocious children may be a sign of mental exhaustion. Rapid action of the heart may also cause some children to appear stupid when such is not the case. Dr. Warner considered that good memory may be merely the faculty of reproducing the record of a series of images or sounds like a phonograph and show no power of adoption, whilst defective memory may be caused by inaccurate observation, itself the result of bad hearing or sight, low health, or deficient nutrition. As an example, let one take some steedlings of the "sensitive plant," plant some in silver sand, some in rich loam. The loam rooted plant becomes callous and bears a blow with equanimity, whilst that rooted in silver sand, fed only on air and water, exhibits extraordinary sensitiveness, the fronds curling up at a breath. A vote of thanks, followed by a short discussion, terminated the proceedings.

### For Reading to the Sick.

### OUR RESPONSIBILITIES.

**Verses.**

Opening the map of God's extensive plan
We find a little isle—this life of man—
Eternity's unknown expanse appears
Circling around, and limiting his years,
The busy race examine and explore
Each creek and cavern of the dangerous shore,
With care collect what in their eyes excels—
Some, shining pebbles, and some, weeds and shells.
A few forsake the throng; and with lifted eyes
Ask wealth of Heaven, and gain a real Prize:
Truth, Wisdom, Grace, and Peace like that above,
Sealed with His signet Whom they serve and love.
Scorned by the rest, with patient hope they wait
A kind release from their imperfect state;
And, unregretted, are soon snatched away
From scenes of sorrow into glorious day. —*Cowper.*

Blaspheme not thou thy sacred Life, nor turn
O'er joys that God hath for a moment lent—
Perchance to try thy spirit and its bent,
Effeminate soul and base—weakly to mourn!
There lies no desert in the land of Life;
For e'en that tract that barrenest doth seem;
Laboured of thee in Faith and Hope, shall teem
With heavenly harvests and rich gatherings rife.
—*Frances Kemble.*

Each hour has its lesson, and each Life;
And if we miss one Life we shall not find
Its lesson in another—rather, go
So much the less complete for evermore,
Still missing something that we cannot name,
Still with our senses so far unattuned
To what the Present brings to harmonise
With our soul's Past. —*H. H. King.*

Lives of great men all remind us
We can make our lives sublime,
And, departing, leave behind us
Footprints on the sands of time.

Footprints that perhaps another,
Sailing o'er Life's solemn main,
A forlorn and shipwreck'd brother,
Seeing, may take heart again. —*Longfellow.*

**Reading.**

I know not a more serious thing than the responsibility incurred by all human affection. Only think of this! Whoever loves you is growing like you. Neither he nor you can hinder it, save at the cost of alienation. Oh, if you are grateful but for one creature's love, rise to the height of so pure a blessing! Drag them not down by the very embrace with which they cling to you, but through their gentleness secure their consecration.

There comes a time to us all when the sense of responsibility starts up and rebukes our anxiety for ease; bids us spring from our collapse of selfishness and sleep, take up the full dimensions of our strength and go forth, to do much—if it is possible—and at least to do worthily and well. No insult can we pass upon the divine but gentle dignity of Duty, no quenching of God's spirit can we allow that will not prepare a curse for others as well as for ourselves; nor any reverence, prompt and due in act as in thought, can we pay to God within that will not yield abundant blessing. —*Martineau.*

### Travel Notes.

By Our Travelling Correspondent.

### IX.—ROCCABRUNA.

Choose a bright, warm morning for an expedition from Nice to Roccabruna; the drive along the upper Corniche is enchanting, but it is somewhat exposed from its great altitude, so that a sunny day is desirable and not much wind. If you are good walkers and able to stand a little fatigue you can reach Roccabruna by train from Nice. It is half-way between Monaco and Mentone, above the beauties of Cap Martin. Leaving the station you mount the heights above abruptly, and a walk of three quarters of an hour lands you in the wonderful little town. The most agreeable way, however, is by carriage from Nice; it is one of the most perfect drives in the world. I have been content myself to make the journey in ramshackle diligences, to which are

Roccabruna.

harnessed three sorry-looking horses, who work well, however, and are by no means in the poor condition they present to the unaccustomed eye. The driving of these public vehicles seems so careless as to fill one with nervous horror; at a narrow part of the road, bounded on one side by immense rocks and on the other by a bottomless pit, it is decidedly critical work to meet an enormous waggon drawn by five mules, stolidly pacing along in the middle of the road, with the driver asleep on the summit of the wine casks, beyond all possibility of being aroused; one holds one's breath and feels one's last hour approach; but no, not this time. In reply to the curses of the diligence driver, who aimably wishes his rival an "accidénte," the mules slowly, and with great dignity and absence of haste, draw to their own side; we hover as it were on the brink of destruction, hurl more curses at the slumberer, and continue our course in all safety. In the drive from Nice you will be put out at the foot of the final climb to Roccabruna, mounting up and up shallow-made steps between stone walls; from here it is quite an easy ascent. The little town is a wonderful specimen of the rock fortress, built as a stronghold against the visits of the Moors. On descrying their ships in the distance the fishermen and peasants hurried to their mountain security. Woe be to those who unwarily tarried behind. I am told that in the beginning of this century there were old men in Mentone and Roccabruna who in their youth had been thus seized and held in slavery by the Moors. In a few appropriate words J. Addington Symonds gives a characteristic account of Roccabruna. "Filled with these Greek fancies it is strange . . . . to enter the village of Roccabruna with its mediæval castle and the motto on its walls, *Tempora labuntur tacitisque senescimus annis.* A true motto for the town where the butcher comes but once a week, and where men and boys and dogs and palms and lemon trees grow up, flourish, and decay in the same hollow of the sunny mountan side. Into the hard conglomerate of the hill the town is built; house walls and precipices mortised into one another, dovetailed by the art of years gone by, and riveted by age. The same plants grow from both alike—spurge, cistus, rue, and henbane, constant to the desolation of abandoned dwellings. From the castle you look down on roofs, brown tiles, and chimney pots, set one above the other like a big card castle. Each house has its foot on its neighbour's neck, and its shoulders set against the native stone. The streets meander in and out and up and down, over-arched and balconied streets, but very clean; they swarm with children, healthy, happy little monkeys who grow fat on salt fish and yellow polenta, and with oil and sun *ad libitum.*"

### The High Street of Roccabruna.

Where everything is a marvel of the picturesque, it is very difficult to make a selection for a sketch, but I finally decided upon taking a view of the principal street, if such it can be called, of Roccabruna. I seated myself under an archway squeezed into the embrasure of a door, to secure myself from the somewhat overbearing manners of the mules, who pace eternally up and down these stairways with enormous panniers slung across their backs, and take their way entirely oblivious of the comfort and safety of such frivolous and useless creatures as tourists and artists. The inhabitants are most friendly and agreeable, and though they collected around me in swarms, were never pushing or rude. Their figures and carriage are most graceful, due probably to the habit of ascending and descending these steep paths with weights on their heads. A little to the left of my sketch is the church, brown and old, though much disfigured inside as usual with tawdry gilding. I wondered, as is my habit, standing in that little isolated sanctuary, how life goes with these simple folk, whether comedy and tragedy goes on here as it does in more sophisticated places

### Lemon Trees.

This part of the Riviera being peculiarly mild, the lemons flourish abundantly. They are far more delicate than oranges, and the slightest approach of frost kills the crop. Round in the hollows about Roccabruna they are sheltered and attain perfection; it is delicious to wander up and down under the old walls and see the rich pale fruit hanging over the grey stones or nestling at the edges of the olive woods. As you descend from the town to take your carriage on the Corniche, the views are superb, the scene is so rich that one feels a desire to have some power of absorbing much more than is possible with one pair of eyes.

La Turbie and Laghetto.

Either going or returning you can visit La Turbie; it stands sullen and forbidding above Monte Carlo. From the tower the view is especially fine. A mile and a half inland stands the ruined convent of Laghetto. I believe you can drive to the very entrance, but having arrived there by a circuitous route on foot from Roccabruna, I am not quite sure.

Here in 1849 Charles Albert, after the disastrous battle of Novara, laid down for ever the symbols of his earthly grandeur, and having devoutly made his confession, left his beloved Italy never to return.

### TRAVEL NOTES AND QUERIES.

Rules in regard to Correspondence for this Section.—All questioners must use a pseudonym for publication, but the communication must also bear the writer's own name and address as well, which will be regarded as confidential. All such communications to be addressed "Travel Editor, 'Nursing Mirror,' 28, Southampton Street, Strand." No charge will be made for inserting and answering questions in the inquiry column, and all will be answered in rotation as space permits. If an answer by letter is required, a stamped and addressed envelope must be enclosed, together with 2s. 6d., which fee will be devoted to the objects of the "Hospital Convalescent Fund." Any inquiries reaching the office after Monday cannot be answered in "The Mirror" of the current week.

Rome (Perplexed).—If your residence will be as long as three or four months it will be advisable to take an apartment, and it is a good plan to rely upon your landlady for attendance, and have your dinners sent in from a trattoria. The portion sent in for dinner is always ample; indeed, one portion is generally enough for two ladies. The water in Rome bears a very good character.

Dinard (Snowdrop).—The English have made it expensive, but St. Servan is still fairly cheap, and is only fifteen minutes from Dinard by ferry steamer. The climate resembles the south of England, not so warm quite, but free from damp and no east winds.

Siena (Bullfinch).—I should not call it quite the place for your case, charming as it is. It is an old walled city, celebrated for the purity of the Italian spoken there. For the artist and archæologist it is unrivalled, and if you can walk and cycle it is delightful, because the surroundings are so fine; but it is not a place to sit out in and indulge day dreams, because it is a town pure and simple, enclosed within walls, and, with the exception of the Lizza, has no public garden.

South of France (Centaur).—I think you would find either Dax or St. Jean-de-Luz, both in the South of France, suitable for your purpose. Dax has a great reputation for its beneficial effects on rheumatism. The cheapest hotel I know of is the Hotel de la Paix, which is 8 francs Pension. For a long stay the proprietor might make still easier terms. St. Jean-de-Luz would be a cheaper and more interesting place; it is on the frontier of Spain. At the Hotel de la Poste they will take you for 7 francs a day Pension, and possibly less by arrangement. If you can speak French you might get a room in the town for very little—5 or 6 francs a week and manage for yourself if money is an object. Write again if I can help you further.

Antwerp for a Week (Quaker).—The return ticket, second class, from London will be £1 4s., and the cheapest hotel that I think you could go to would be Hotel du Commerce, in the Rue de la Bourse. Pension 7 francs a day, which is 6s. 8d. You must not think of taking a room. Antwerp is not at all the place in which to run any risks, nor would it be an economy for one week. Seven days at 7 francs will be £2 16s., your hotel tips will be 4 francs, making another 3s. 6d. You can therefore do it comfortably for £5, having 16s 6d. left for trams, omnibuses, and food on the journeys. Ask again if I can tell you anything more.

*For Travel Advertisements see page* xxii.

### Presentations.

Miss Eleanor J. Law, who some months ago resigned the post of lady superintendent and matron of the Royal Berks Hospital, Reading, and who is leaving about the middle of the month, has had several charming gifts from the nursing staff and servants. The nurses gave her a silver card case with her initials engraved upon it, as well as a silver-mounted purse; the women servants and housekeeper a china and silver biscuit box; and the men servants and porters a silver serviette ring.

Miss Geard, who has recently resigned her post of matron to the Ear and Throat Hospital, Birmingham, on account of her approaching marriage, has been the recipient of many presents, including a purse of money from the committee of the hospital, a Davenport from the nurses past and present, a clock from the servants, and table silver from some of the inhabitants of Marazion, Cornwall, where she was working for upwards of two years.

### Notes and Queries.

The contents of the Editor's Letter-box have now reached such unwieldy proportions that it has become necessary to establish a hard and fast rule regarding Answers to Correspondents. In future, all questions requiring replies will continue to be answered in this column without any fee. If an answer is required by letter, a fee of half-a-crown must be enclosed with the note containing the enquiry. We are always pleased to help our numerous correspondents to the fullest extent, and we can trust them to sympathise in the overwhelming amount of writing which makes the new rules a necessity.

Every communication must be accompanied by the writer's name and address, otherwise it will receive no attention.

*Training near London.*

(187) I am desirous of entering an infirmary as probationer, preferably in the county of Hertford, or country near London. Can you tell me where to apply? I would require a salary.—*Hertford.*

See a new book just issued by the Scientific Press (London, W.C.), "The Nursing Profession: How and Where to Train," price 2s.

*Stewardesses.*

(188) Will you kindly give me some information respecting the duties, salaries, &c., of stewardesses on ships carrying passengers? I and a friend are anxious to obtain posts together, but do not know how to set about it. We both hold three-year certificates for general training, also one for infectious work.—*Anxious.*

The only way is to apply to the secretaries of the shipping companies (see advertisements in daily papers for addresses). There is difficulty in getting these posts, and there is sometimes a long wait for a vacancy.

*Surgical Dressings.*

(189) A surgical nurse would be glad to know whether the correct way to apply lotio rubra is by soaking lint two thicknesses in the lotion, cutting it the exact size of the wound, and covering it with gutta-percha tissue cut a little larger than the lint; also the name of some good work on surgical dressings.—*Nurse Lamming.*

When the lotion is intended to be applied after the manner of the old fashioned water dressing, the lint should be cut the size of the wound, and the guttapercha as much larger than the lint as will prevent evaporation. 2. "Surgical Ward Work," by Miles, would probably suit you (Scientific Press, 3s. 6d.)

*Permanent Home.*

(190) Can anyone tell me of a small Home where a middle-aged woman—asthmatical—could be received for an unlimited time by payment of from 5s. to 7s. per week? Seaside preferred.—*E. P.*

"Holiday Homes," &c., price 1½d., from Miss Cole, 188, Ebury Street, S.W., gives an excellent choice

*Training.*

(191) Will you kindly advise me as to the best provincial eye hospital at or near the sea for a first training? Have the Bristol or Sunderland Eye Hospitals good training? One with all non-paying lady probationers preferred, and small salary first year.—*I. C. T.*

Your best plan is to consult "The Nursing Profession," recently issued by the Scientific Press, price 2s. There you will find a full list of all the training schools and the terms offered.

*Holiday*

(192) Can you tell me if there is a holiday fund for nurses where a nurse could have a child with her for a fortnight?—*Probationer.*

There is no special fund to provide nurses with holidays. See list of "Holiday Homes," &c., post free 1½d., from Miss Cole, 188, Ebury Street, S.W.

*Mental Training.*

(193) Will you kindly tell me (1) the name of a good asylum where there is training given for mental nurses? 2 The length of training required in order to gain the psychological certificate? 3. What is the age limit, if any, for the training? 4. Is it likely one could always get work and a good salary if certificated?—*R. L. M.*

The Northampton County Asylum, Berrywood, and several others are good training schools for mental nurses. (See "The Nursing Profession," price 2s., from the Scientific Press, London.) 2. Two years until October, when a three years' period will be substituted. 3. There is no definite age limit. 4. Yes, a good mental nurse is sure of employment and good pay if she is reliable and strong.

*Dispensing.*

(194) How can I learn dispensing, and what examinations must I pass? I do all my own dispensing here, but seek further knowledge.—*Cottage Hospital Matron,*

You must prepare for the assistant examinations of the Apothecaries' Hall, Blackfriars, S.W. Apply to the secretary for particulars.

*A Cottage Nurse.*

(195) Can you tell me where I could hear of a cottage nurse for a country village? I have applied at the Nurses' Home, Plaistow, but can hear of no one willing to undertake the ordinary work of a cottage as well as nursing.—*M. B.*

Your difficulty illustrates the inner working of the system of cottage nursing. As soon as a woman receives a modicum of training, and is free from her agreement, she competes with the fully-trained nurse, and refuses to accept the domestic duties which are the excuse for her existence. We doubt very much your being able to get one unless you pay for her training.

*Epileptics.*

(196) Will the editor kindly give "District Nurse" the addresses of the three homes for epileptics, and any information which would help her to get a young man into one?

Home for Epileptics, Maghull, near Liverpool; terms of admission by payment of 7s. 6d. to £2 2s. and upwards a week. National Society for the Employment of Epileptics, Chalfont St. Peter's. Apply the Secretary, 12, Buckingham Street, Strand. The Meath Home at Godalming is for young women.

## Figures and Tables

**Figure f1:**